# Revisiting One-Carbon Metabolites in Human Breast Milk: Focus on S-Adenosylmethionine

**DOI:** 10.3390/nu15020282

**Published:** 2023-01-05

**Authors:** Carles Lerin, María Carmen Collado, Elvira Isganaitis, Erland Arning, Brandi Wasek, Ellen W. Demerath, David A. Fields, Teodoro Bottiglieri

**Affiliations:** 1 Endocrinology Department, Institut de Recerca Sant Joan de Déu, Hospital Sant Joan de Déu, 08950 Barcelona, Spain; 2 Centro de Investigación Biomédica en Red de Diabetes y Enfermedades Metabólicas Asociadas (CIBERDEM), Instituto de Salud Carlos III, 28029 Madrid, Spain; 3 Department of Biotechnology, Institute of Agrochemistry and Food Technology-National Research Council (IATA-CSIC), 46980 Valencia, Spain; 4 Research Division, Joslin Diabetes Center, Harvard Medical School, Boston, MA 02215, USA; 5 Center of Metabolomics, Institute of Metabolic Disease, Baylor Scott and White Research Institute, Dallas, TX 75204, USA; 6 Division of Epidemiology and Community Health, The University of Minnesota School of Public Health, Minneapolis, MN 55455, USA; 7 Department of Pediatrics, University of Oklahoma Health Sciences Center, Oklahoma City, OK 73104, USA

**Keywords:** breast milk, breastfeeding, milk formula, one-carbon metabolism, S-adenosylmethionine

## Abstract

Breastfeeding is the gold standard for early nutrition. Metabolites from the one-carbon metabolism pool are crucial for infant development. The aim of this study is to compare the breast-milk one-carbon metabolic profile to other biofluids where these metabolites are present, including cord and adult blood plasma as well as cerebrospinal fluid. Breast milk (*n* = 142), cord blood plasma (*n* = 23), maternal plasma (*n* = 28), aging adult plasma (*n* = 91), cerebrospinal fluid (*n* = 92), and infant milk formula (*n* = 11) samples were analyzed by LC-MS/MS to quantify choline, betaine, methionine, S-adenosylmethionine, S-adenosylhomocysteine, total homocysteine, and cystathionine. Differences between groups were visualized by principal component analysis and analyzed by Kruskal–Wallis test. Correlation analysis was performed between one-carbon metabolites in human breast milk. Principal component analysis based on these metabolites separated breast milk samples from other biofluids. The S-adenosylmethionine (SAM) concentration was significantly higher in breast milk compared to the other biofluids and was absent in infant milk formulas. Despite many significant correlations between metabolites in one-carbon metabolism, there were no significant correlations between SAM and methionine or total homocysteine. Together, our data indicate a high concentration of SAM in breast milk, which may suggest a strong demand for this metabolite during infant early growth while its absence in infant milk formulas may indicate the inadequacy of this vital metabolic nutrient.

## 1. Introduction

The composition of breast milk has elegantly evolved to meet the specific requirements of developing organisms. From nutrients to hormones to immune system components, oligosaccharides, and specific microorganisms, breast milk contains a wide range of molecules that serve as the unique nutritional source required for healthy growth and development [1]. When breastfeeding is not feasible, milk substitutes have been developed to meet the nutritional requirements of infants. However, the optimal composition to promote healthy infant growth may require further refinement. Compared to artificial milk formula use, breastfeeding has been consistently associated with a lower risk of infections, diarrhea, allergies, or obesity [1,2]. Consequently, exclusive breastfeeding during the first 6 months of age is strongly recommended by major public health agencies including the World Health Organization [1,3].

**Figure 1:**
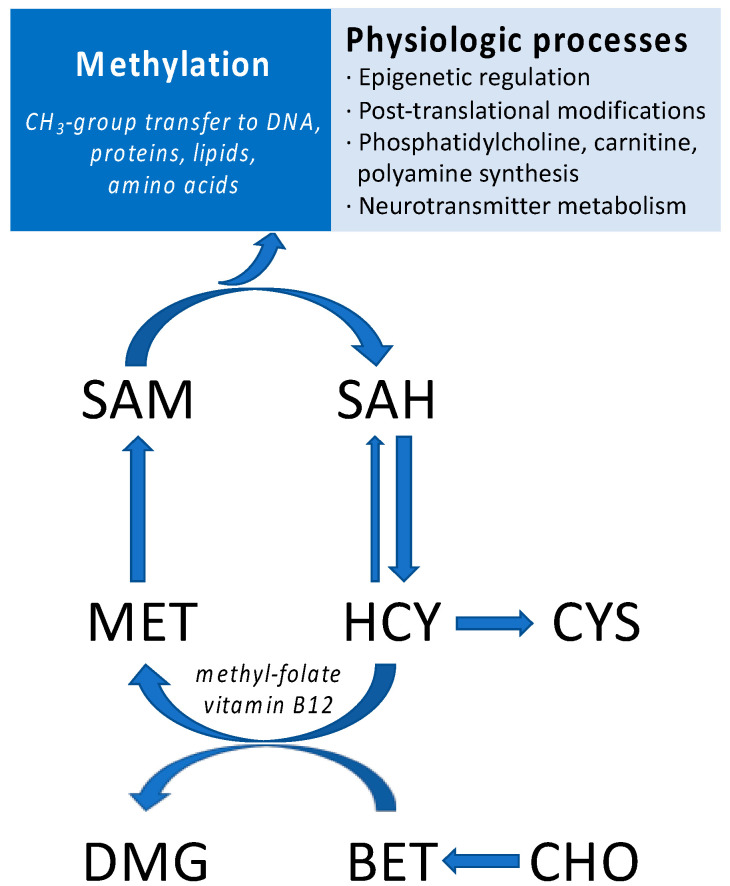
Schematic representation of one-carbon metabolism-associated pathways. CHO, choline; BET, betaine; MET, methionine, SAM, S-adenosylmethionine; SAH, S-adenosylhomocysteine; CYS, cystathionine; HCY, homocysteine.

Among the milk bioactive molecules, metabolites from the one-carbon metabolism pool are important nutrients for infant development and participate in crucial physiologic processes (Figure 1) [4,5]. For instance, breast milk is a rich source of choline, an essential nutrient for infant development [6,7]. Choline is used as a substrate in several important reactions, including neurotransmitter and phosphatidylcholine biosynthesis, or can also be oxidized to betaine. In mammals, betaine is primarily metabolized in the liver, where it can transfer a methyl group to homocysteine to synthesize methionine. Subsequent enzymatic reactions convert methionine into S-adenosylmethionine (SAM), S-adenosylhomocysteine (SAH), and homocysteine in the so-called methionine cycle (Figure 1; for review, see [8]). SAM is an important metabolite that participates in multiple critical reactions for infant development, including phosphatidylcholine, polyamine, and carnitine biosynthesis, or DNA and protein methylation [8]. SAM is also available in several countries as an over-the-counter dietary supplement, which is supported by numerous clinical trials indicating its efficacy in the treatment of a wide range of conditions, including depression [9,10], hepatic disorders [11,12], and osteoarthritis [13,14].

We recently performed one-carbon metabolite profiling in human milk samples from two independent cohorts [15]. In this study, we observed remarkably high concentrations of SAM in this biofluid compared to levels reported in plasma in the literature [16,17]. Given the lack of previous reports of the SAM content in breast milk and the fundamental role that SAM plays in cellular biology, we sought to compare levels of metabolites related to one-carbon metabolism, with a focus on SAM, in human breast milk with other biofluids where these metabolites are present, including cord blood, maternal plasma, healthy aging adult plasma, and cerebrospinal fluid (CSF). In addition to the biofluids, we tested several infant milk formulas as possible sources of one-carbon nutrients in formula-fed infants.

## 2. Materials and Methods

### 2.1. Study Design

Human samples used in the present study originated from previous studies: (1) breast milk samples from two independent cohorts (34 samples were from the US-based MILK study and 109 samples from the European-based MAMI cohort) were obtained at 1 month after birth [15,18]; (2) maternal and cord blood plasma (*n* = 28 and *n* = 23, respectively) were obtained at delivery from the MAMI cohort [18]; (3) blood plasma and CSF (*n* = 91 and *n* = 92, respectively) from cognitively normal adults were obtained from longitudinal studies of healthy aging and dementia at the Knight Alzheimer’s Disease Research Center at Washington University in St. Louis, MO, USA. Demographic characteristics of participants are reported in Supplementary Table S1. All samples were from normal healthy individuals. The studies were conducted in accordance with the Declaration of Helsinki, the protocols were approved by the corresponding official institutional review board, and informed consent was obtained from all participants. Milk formulas (*n* = 11) were commercially available (seven purchased from local stores in the US and four obtained from different pharmacies in Spain). All milk formulas were suitable for newborn infants (milk formulas appropriate for infants between 0 and 6 months of age).

### 2.2. Metabolite Analysis

All biofluid samples were stored at −80 °C until analysis. Eleven commercially available milk formulas for infants younger than 6 months were analyzed in triplicate. Choline, betaine, methionine, SAM, SAH, and cystathionine were determined by liquid chromatography coupled with mass spectrometry (LC-MS/MS) as previously described [17]. This analysis in human breast milk samples had been previously reported [15]. Briefly, breast milk samples were processed by ultrafiltration utilizing the microcentrifugal filter units Microcon YM-10 and 10 kDa NMWL (Millipore, Burlington, MA, USA) prior to LC-MS/MS analysis. For formula milk, each product was weighed and dissolved in the corresponding volume of distilled water following the manufacturer’s instructions. When the milk powder was added to the solution, it was further diluted for metabolite determination, and an aliquot of 50 µL was used for LC-MS/MS analysis of metabolites as described for breast milk samples. The total homocysteine (tHCY) was measured by HPLC-fluorescence in CSF as previously described [19] and by LC-MS/MS in breast milk and plasma samples [16]. Briefly, samples were prepared by adding 10 µL of breast milk, plasma, or standards to 120 µL of internal standard solution (containing d4-homocysteine in 4 mM of dithiothreitol dissolved in distilled water). After incubation at room temperature for 30 min, samples were deproteinized with 200 µL acetonitrile and 0.1% formic acid and centrifuged at 1400 rpm for 5 min. Samples were analyzed following the injection of 10 µL of extract on a Synergi Hydro 4 µ 150 × 3 mm (Phenomenex), maintained at 40 °C, and eluted in a gradient with buffer A (100% water, 0.5% formic acid and 0.25% heptafluorobutyric acid) and buffer B (100% acetonitrile, 0.5% formic acid 0.25% heptafluorobutyric acid). The flow rate was 0.5 mL/min, with a step-wise gradient over a total run time of 10 min. Mass spectrometry was performed on a 5500 QTrap (Sciex, Framingham, MA, USA), and the observed (*m*/*z*) values of the fragment ions were homocysteine (*m*/*z* 136 → 90) and d4-homocysteine (*m*/*z* 140 → 94). All data were collected and processed using Analyst software v1.4.2 (Sciex, Framingham, MA, USA). The coefficient of variation for all metabolites between assays had a range of 6.2–17.8%. The limits of detection and calibration curves are reported in Table S2. One milk sample was identified as an outlier due to a high tHCY concentration (20.5 µmol/L) and removed from the analysis.

### 2.3. Statistical Analysis

Unless otherwise stated, descriptive data are shown as the median and interquartile range. Data normality was assessed with the Shapiro–Wilk test. Data were log-transformed before principal component analysis (PCA) was performed. Differences between groups were assessed with the non-parametric Kruskal–Wallis test and post hoc Dunn method, with Bonferroni correction to account for multiple comparisons. The correlation of one-carbon metabolites in human breast milk was performed on log-transformed concentration data using the REML method to calculate the intra-class correlation between each metabolite. Correlations are reported as the Pearson correlation coefficient with the corresponding *p*-value. A two-tailed *p*-value below 0.05 was considered statistically significant. Statistical analyses were performed in JMPv16 (SAS Institute Inc., Cary, NC, USA).

## 3. Results

**Figure 2:**
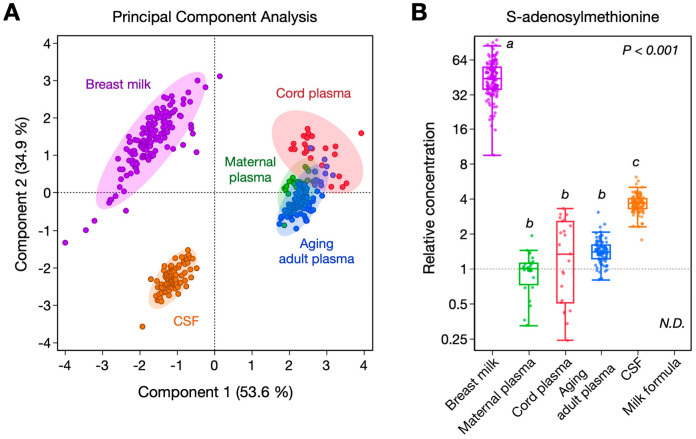
One−carbon metabolite profiling in biofluids and milk formula. One-carbon metabolite concentrations were measured in breast milk (purple, *n* = 142), cord blood plasma (red, *n* = 23), maternal plasma (green, *n* = 28), aging adult plasma (blue, *n* = 91), CSF (orange, *n* = 92), and milk formula (*n* = 11). (**A**) PCA score plot based on metabolite concentrations in the different biofluids; shaded areas show 95% confidence regions. (**B**) Boxplot of SAM concentration in biofluids and milk formula relative to the median values for the maternal plasma samples shown with logarithmic Y-axis. Kruskal–Wallis test with post hoc Dunn method was applied; groups not connected by the same letter show statistical differences (*p* < 0.05). CHO, choline; BET, betaine; MET, methionine, SAM, S-adenosylmethionine; SAH, S-adenosylhomocysteine; CYS, cystathionine; tHCY, total homocysteine; CSF, cerebrospinal fluid; N.D., not detected.

Absolute concentrations for the one-carbon metabolites in the different biofluids are shown in Table 1. The PCA score plot based on the seven metabolites analyzed showed a clear separation of milk samples from other biofluids, with the different types of plasma samples (cord blood, maternal, and healthy aging adult plasma) clustering together (Figure 2A). To compare metabolite concentrations across biofluids, we used maternal plasma median values as a reference. The SAM content was strikingly elevated in breast milk, reaching a median concentration of 1830 nmol/L (Table 1), 44-fold higher than maternal plasma values (median concentration of 42 nmol/L, Table 1 and Figure 2B). In comparison, the SAM concentration in cord blood, aging adult plasma, and CSF was 1.3-, 1.4-, and 3.7-fold higher, respectively, compared to maternal plasma (Table 1, Figure 2B). Choline and SAH concentrations were also higher in breast milk (10.5- and 6.4-fold, respectively) compared to maternal plasma (Table 1). Conversely, the betaine, methionine, cystathionine, and homocysteine concentrations were 0.3-, 0.2-, 0.5-, and 0.1-fold lower, respectively (Table 1). Notably, SAM was undetected in all of the infant milk formulas analyzed, while other metabolites, especially choline and methionine, were present at higher concentrations than in human breast milk (Table 1).

Multivariate correlation coefficients between each metabolite in human breast milk and the corresponding probability values are shown in Table 2. As may be expected, many metabolites are significantly correlated in the one-carbon metabolism pathway. Of interest is the absence of a significant correlation between SAM and its substrate precursor metabolite methionine and demethylated metabolite tHCY.

## 4. Discussion

In this study, we compared the levels of one-carbon metabolites in breast milk with other biofluids where these metabolites are present, including plasma and CSF. We show that breast milk has a distinct one-carbon metabolic profile than other biofluids, including cord blood and maternal plasma, healthy aging adult plasma, and CSF. The concentrations of some of these metabolites in breast milk have been previously reported [6,7,20,21]. For instance, it is well known that choline is substantially higher in milk than in plasma, indicating a key role of this nutrient in infant development; consistent with other studies [7], we found a 10-fold increase in milk choline compared to plasma.

In recent years, several studies have applied metabolomic approaches to further investigate breast milk composition [22,23,24,25]. However, to our knowledge, there are no previous reports of SAM concentration in breast milk, with the exception of our previous report in the same cohort [15]. Our study demonstrates that the SAM concentration is significantly higher in breast milk compared to the other biofluids, with a 44-fold increase compared to maternal plasma and 12-fold increase compared to CSF (see Table 1 and Figure 2B). The SAM levels in maternal and adult plasma are not significantly different, suggesting that the high SAM content observed in milk is derived from breast tissue and not circulating plasma (see Table 1). SAM is the methyl-group donor for most transmethylation reactions in the organism, including phosphatidylcholine, polyamine, and carnitine biosynthesis, as well as DNA and protein methylation [26]. The by-product metabolite of methylation is SAH. Interestingly, the SAH levels in breast milk are approximately 6-fold higher than maternal and cord blood (see Table 1), which likely reflects a higher activity of methylation reactions.

Since SAM is the principal methyl-group donor in multiple cellular methyltransferase pathways and given its high concentration in breast milk, it is tempting to speculate that SAM may be a crucial nutrient in infant growth and development and may play an essential role during early stages of life. There is growing evidence to suggest that epigenetic mechanisms, specifically DNA methylation, including posttranslational histone modifications, take place early in development and could persist well into later stages of life that are associated with disease in adulthood [27]. In particular, DNA methylation modifications in genes regulating the hypothalamus pituitary adrenal axis and the immune system have been identified in infants that are related to cardiometabolic disease [28]. Another recent study has determined DNA methylation changes in saliva samples during the first year of life. Clear differences in DNA methylation were found between 6 and 52 weeks of age in 42 genes; 36 genes showed increased, and 6 genes showed decreased DNA methylation [29]. It has been suggested that the increased methylation, which is associated with overall decreased gene expression, may represent a slowing mechanism to reduce extensive growth development following the period of extremely rapid growth during pregnancy. The full extent of DNA methylation on specific genes during infancy and childhood development is still not fully understood. However, SAM as a methyl-donor in breast milk may play an important role in this respect. It is required for the methylation of phospholipids, more specifically the synthesis of phosphatidylcholine that can be recycled to choline, an important metabolite in breast milk for neurodevelopment.

It is important to note that SAM is absent in the different milk formulas analyzed in this study (see Figure 2B). Formula-fed infants can hydrolyze proteins to obtain methionine and convert it into SAM. Although methionine is present in a high concentration in formula milk and can be converted to SAM, this occurs mostly in hepatic tissue where several isoforms of methionine adenosyltransferase (MAT) with a wide Km range are present. Other tissues including central nervous system tissue have a limited capacity to convert methionine to SAM since only a single low-Km MAT isoform is present [30]. This may be critical for neurodevelopment and assessing the plasma levels of metabolites related to one-carbon metabolism in breast-milk-fed and formula-fed infants would be required to further our understanding of the importance of elevated SAM levels in breast milk. It is also important to note that the concentration of SAM in breast milk is close to peak plasma concentrations following oral administration of enteric-coated SAM tablets. In one study in women, the SAM levels reached a C_max_ of 2.5 μM at 5.2 h following an oral dose of 1000 mg SAM-tosylate [31]. In our study, we found that the level of SAM in breast milk is 1.83 μM. This may provide some assurance that levels of breast milk SAM may not increase substantially after oral supplementation. However, further studies are required to determine if repeated chronic oral supplementation of SAM results in accumulation and higher levels in breast milk. This is particularly important as breastfeeding mothers may use oral SAM supplementation to prevent post-partum depression, as standard antidepressant therapies are viewed as being harmful to the infant [32].

Limitations of this study include the fact that breast milk samples were obtained at a single time point (1 month after birth). As composition varies with time, longitudinal studies of one-carbon metabolism in breast milk are required to assess the dynamic changes during the critical period of nutritional support in infants.

## 5. Conclusions

S-adenosylmethionine is particularly elevated in breast milk compared to other biofluids. High concentrations of SAM in breast milk highlight the need to further investigate the role of this metabolite in breast milk as a readily available micronutrient for newborns and opens the venue for revisiting milk formula composition.

## Figures and Tables

**Table 1:** One-carbon metabolite concentrations in biofluids and milk formula.

	Breast Milk (*n* = 142)	Maternal Plasma (*n* = 28)	Cord Plasma (*n* = 23)	Adult Plasma (*n* = 91)	Cerebrospinal Fluid (*n* = 92)	Milk Formula (*n* = 11)	*p* Value
CHO, µmol/L	123.5 (92.8) ^a^	11.8 (4.2) ^b^	56.8 (36.0) ^a,b^	7.1 (2.2) ^b^	2.7 (1.0) ^c^	1510 (208) ^d^	<0.0001
BET, µmol/L	3.6 (3.2) ^a^	12.2 (5.5) ^b^	30.9 (16.7) ^b,c^	35.9 (14.8) ^c^	1.9 (0.6) ^d^	16.0 (15.7) ^a,b^	<0.0001
MET, µmol/L	4.2 (2.1) ^a^	21.4 (8.6) ^b,c^	31.0 (5.7) ^b^	21.1 (4.5) ^b,c^	3.2 (1.0) ^d^	272 (358) ^c^	<0.0001
SAM, nmol/L	1830 (805) ^a^	42 (16) ^b,c^	56 (86) ^b,c^	59 (16) ^b^	154 (29) ^d^	<0.1 ^c^	<0.0001
SAH, nmol/L	263 (192) ^a^	41 (14) ^b^	43 (39) ^b,c^	26 (8) ^b,c^	10 (6) ^d^	18.6 (27.7) ^c,d^	<0.0001
tHCY, µmol/L *	0.27 (0.15) ^a^	7.38 (2.60) ^b^	7.05 (2.03) ^b^	5.70 (2.90) ^b^	0.09 (0.03) ^c^	N.A.	<0.0001
CYS, µmol/L	91 (91) ^a^	199 (107) ^b^	334 (187) ^b^	105 (61) ^a^	43 (20) ^c^	20.9 (11.3) ^c^	<0.0001

Data are shown as median (interquartile range). Differences between groups were assessed with the Kruskal–Wallis test with the post hoc Dunn method; groups not connected by the same letter show statistical differences (*p* < 0.05). CHO, choline; BET, betaine; MET, methionine, SAM, S-adenosylmethionine; SAH, S-adenosylhomocysteine; CYS, cystathionine; tHCY, total homocysteine. *, data from 138 breast milk samples; N.A., not analyzed. CHO, BET, MET, SAM, SAH, and CYS concentrations in breast milk were previously reported in reference [15].

**Table 2:** One-carbon metabolite correlations in breast milk samples.

	CHO	BET	MET	SAM	SAH	CYS	tHCY
CHO	1						
BET	0.58 (<0.001)	1					
MET	0.43 (<0.001)	0.25 (0.003)	1				
SAM	0.01 (0.904)	−0.18 (0.035)	0.02 (0.851)	1			
SAH	0.25 (0.003)	0.45 (<0.001)	0.09 (0.262)	−0.20 (0.015)	1		
CYS	0.46 (<0.001)	0.45 (<0.001)	0.32 (<0.001)	0.32 (<0.001)	0.42 (<0.001)	1	
tHCY *	0.30 (<0.001)	0.22 (0.009)	0.25 (0.003)	0.08 (0.376)	0.26 (0.002)	0.41 (<0.001)	1

Data are shown as Pearson correlation coefficients with their corresponding *p* values from log-transformed concentrations of the one-carbon metabolites in breast milk samples (*n* = 142). CHO, choline; BET, betaine; MET, methionine, SAM, S-adenosylmethionine; SAH, S-adenosylhomocysteine; CYS, cystathionine; tHCY, total homocysteine. *, data from 138 samples.

## Data Availability

The data presented in this study are available on request from the corresponding author. The data are not publicly available due to ethical reasons arising from the different studies from which they originated.

## Supplementary Materials

The following supporting information can be downloaded at: https://www.mdpi.com/article/10.3390/nu15020282/s1, Table S1: Assay performance of LC-MS/MS method and calibration curves used for samples analyzed; Table S2: Demographic characteristics of participants.
