# Supplementary material for: Revisiting One-Carbon Metabolites in Human Breast Milk: Focus on S-Adenosylmethionine
Source: Nutrients. 2023 Jan 5;15(2):282. doi: 10.3390/nu15020282 (PMC9863976; doi:10.3390/nu15020282)
Supplement: Supplementary file 1 [file nutrients-15-00282-s001.zip › nutrients-2086512-supplementary.pdf]

SUPPLEMENTARY INFORMATION

Table S1. Demographic characteristics of participants.

	Breast milk [1] (n=142)	Maternal plasma (n=28)	Cord blood (n=23)	Adult plasma (n=91)	CSF (n=92)
Gender (F/M)	-	-	-	66/25	67/25
Age (years)	31 (28,37)	36 (32,38)	36 (32,38)	60 (51,72)	60 (51,72)
Pregestational BMI	23.0 (21.0,26.6)	23.0 (19.6,26.5)	23.4 (20.2,26.0)	-	-
GWG (kg)	12 (9,15) [2]	12 (9,15)	12 (10,16)	-	-
Infant gender (F/M)	74/68	14/14	6/17	-	-
Delivery method (V/C)	91/51	14/14	6/17	-	-
Gestational age (weeks)	40 (39,40)	39 (38,40)	38 (38,39)	-	-
Birth weight (kg)	3.3 (3.1,3.7)	3.1 (2.9,3.7)	2.9 (2.7,3.4)	-	-

Data are shown as median with 25th and 75th quartiles. CSF, cerebrospinal fluid; BMI, body mass index; GWG, gestational weight gain; F, female; M, male; V, vaginal; C, C-section.

[1] Pooled data from two cohorts (details for each cohort can be found in reference 15).

[2] Data for 140 subjects.

Table S2. Assay performance of LC-MS/MS method and calibration curves used for samples analyzed.

Between assay Coefficient of Variation (%) Using QC 1

	tHcy	MET	SAM	SAH	BET	CHO	CYSTA (µM)
Plasma	7.9	6.9	12.2	11.4	17.8	6.2	7.5

Upper Limit of Quantitation (ULOQ) Without dilution

	tHcy (µM)	MET (µM)	SAM (µM)	SAH (µM)	BET (µM)	CHO (µM)	CYSTA (µM)
Plasma	500	200	10	10	200	600	10

Lower Limit of Quantitation (LLOQ)

	tHcy (µM)	MET (µM)	SAM (nM)	SAH (nM)	BET (µM)	CHO (µM)	CYSTA (nM)
Plasma	0.8	5	5	5	5	1	25

Plasma calibration curve

	tHcy (µM)	MET (µM)	SAM (nM)	SAH (nM)	BET (µM)	CHO (µM)	CYSTA (nM)
Blank	0	0	0	0	0	0	0
Cal 1	2.5	6.25	25	25	6.25	6.25	0.625
Cal 2	5	12.5	50	50	12.5	12.5	1.25
Cal 3	10	25	100	100	25	25	2.5
Cal 4	20	50	200	200	50	50	5
Cal 5	40	100	400	400	100	100	10
Cal 6	80	NA	NA	NA	NA	NA	NA

CSF calibration curve

	tHcy*	MET (µM)	SAM (nM)	SAH (nM)	BET (µM)	CHO (µM)	CYSTA (nM)
Blank	NA	0	0	0	0	0	0
Cal 1	NA	0.625	25	25	0.625	0.625	0.625
Cal 2	NA	1.25	50	50	1.25	1.25	1.25
Cal 3	NA	2.5	100	100	2.5	2.5	2.5
Cal 4	NA	5	200	200	5	5	5
Cal 5	NA	10	400	400	10	10	10

Breast Milk calibration curve

	tHcy (µM)	MET (µM)	SAM (nM)	SAH (nM)	BET (µM)	CHO (µM)	CYSTA (nM)
Blank	0	0	0	0	0	0	0
Cal 1	62.5	0.5	25	25	0.5	6.25	0.625
Cal 2	125	1	50	50	1	12.5	1.25
Cal 3	250	2	100	100	2	25	2.5
Cal 4	500	4	200	200	4	50	5
Cal 5	1000	8	400	400	8	100	10

Milk Formula calibration curve

	tHcy	MET (µM)	SAM (nM)	SAH (nM)	BET (µM)	CHO (µM)	CYSTA (nM)
Blank	ND	0	0	0	0	0	0
Cal 1	ND	0	0	0	0	0	0
Cal 2	ND	6.25	25	25	6.25	6.25	0.625
Cal 3	ND	12.5	50	50	12.5	12.5	1.25
Cal 4	ND	25	100	100	25	25	2.5
Cal 5	ND	50	200	200	50	50	5
Cal 6	ND	100	400	400	100	100	10

NA, not applicable; ND, not determined. * CSF tHcy was determined by HPLC-fluorescence as previously described (reference [19]). The method was adapted for analysis of CSF samples using a calibration curve from 50 - 2000 nM. CHO, choline; BET, betaine; MET, methionine, SAM, S-adenosylmethionine; SAH, S-adenosylhomocysteine; CYS, cystathionine; tHcy, total homocysteine.

HPLC-fluorescence
